# miR-24 Targets the Transmembrane Glycoprotein Neuropilin-1 in Human Brain Microvascular Endothelial Cells

**DOI:** 10.3390/ncrna7010009

**Published:** 2021-02-02

**Authors:** Pasquale Mone, Jessica Gambardella, Xujun Wang, Stanislovas S. Jankauskas, Alessandro Matarese, Gaetano Santulli

**Affiliations:** 1 Department of Medicine, Division of Cardiology, Wilf Family Cardiovascular Research Institute, Einstein Institute for Aging Research, Albert Einstein College of Medicine, New York, NY 10461, USA; pasqualemone@hotmail.it (P.M.); jessica.gambardella@einsteinmed.org (J.G.); xujun.wang@einsteinmed.org (X.W.); stanislovas.jankauskas@einsteinmed.org (S.S.J.); 2 Department of Advanced Medical and Surgical Sciences, University of Campania “*Luigi Vanvitelli*”, 80100 Naples, Italy; 3 Department of Molecular Pharmacology, Einstein-Mount Sinai Diabetes Research Center (*ES-DRC*), Fleischer Institute for Diabetes and Metabolism (*FIDAM*), Albert Einstein College of Medicine, New York, NY 10461, USA; 4 Department of Advanced Biomedical Science, “Federico II” University, and International Translational Research and Medical Education (ITME), 80131 Naples, Italy; 5 AORN “*Antonio Cardarelli*”, 80100 Naples, Italy; alessandromatarese@yahoo.it

**Keywords:** ACE2, Akt, blood–brain barrier, brain, coronavirus, CD304, COVID-19, endothelium, epigenetics, hBMEC, microRNA, miR-24-3p, neurology, non-coding RNA, NRP1, SARS-CoV-2, vascular permeability, VEGF, VEGF165R

## Abstract

Neuropilin-1 is a transmembrane glycoprotein that has been implicated in several processes including angiogenesis and immunity. Recent evidence has also shown that it is implied in the cellular internalization of the severe acute respiratory syndrome coronavirus (SARS-CoV-2), which causes the coronavirus disease 2019 (COVID-19). We hypothesized that specific microRNAs can target Neuropilin-1. By combining bioinformatic and functional approaches, we identified miR-24 as a regulator of Neuropilin-1 transcription. Since Neuropilin-1 has been shown to play a key role in the endothelium-mediated regulation of the blood-brain barrier, we validated miR-24 as a functional modulator of Neuropilin-1 in human brain microvascular endothelial cells (hBMECs), which are the most suitable cell line for an in vitro blood–brain barrier model.

## 1. Introduction

Neuropilins are single-pass transmembrane, non-tyrosine kinase surface glycoproteins that are expressed in all vertebrates with versatile roles in a wide range of physiological processes including angiogenesis, immunity, development, and axonal guidance [1,2,3,4,5]. The family includes two homologous isoforms, Neuropilin-1 and Neuropilin-2, encoded by distinct genes on different chromosomes (10p12 and 2q34, respectively) [6]. Both isoforms are upregulated in a number of clinical disorders, including cancer, where they increase the oncogenic activities of malignant cells by promoting survival, inducing angiogenesis and lymphangiogenesis, and contribute to therapy resistance [7]. Neuropilin-1 has been shown to regulate the endothelium-dependent inflammatory responses at the level of the blood–brain barrier [8].

MicroRNAs (miRNAs, miRs) are small non-coding RNAs involved in post-transcriptional gene regulation [9,10,11,12]. They play crucial regulatory roles in a number of biological processes [13,14,15,16,17,18,19,20]. Of note, miRNAs represent a very attractive therapeutic strategy to manipulate various processes as their activity can be efficiently modulated with innovative and personalized technologies [21,22]. We, and others, have identified a number of miRNAs involved in the regulation of endothelial function [23,24,25,26,27,28].

The main aim of this study was to identify miRNAs that specifically target Neuropilin-1 in human brain endothelial cells. We were able to pinpoint and validate hsa-miR-24-3p (indicated for brevity as miR-24) as a main regulator of Neuropilin-1 transcription.

## 2. Materials and Methods

### 2.1. Cell Culture and Reagents

All reagents were purchased from Millipore-Sigma (Burlington, MA, USA), unless otherwise stated. Human brain microvascular endothelial cells (hBMECs) were obtained from Neuromics (Minneapolis, MN, USA; catalog number: #HEC02). These cells have been proved to be the most suitable human cell line for an in vitro blood–brain barrier (BBB) model [29].

Cells were cultured in a standard humidified atmosphere (37 °C) containing 5% CO_2_. In some experiments, cells were transfected with pcDNA3.1-*Neuropilin-1* plasmids (GenScript, Piscataway, NJ, USA).

### 2.2. Identification of miRNAs Targeting Neuropilin-1

To identify miRNAs targeting the 3′-UTR of Neuropilin-1, we used the online target prediction tool Targetscan 7.2, as previously described by our research group [28,30,31,32,33].

### 2.3. Biological Validation of miR-24 as a Regulator of Neuropilin-1

**Figure 1:**
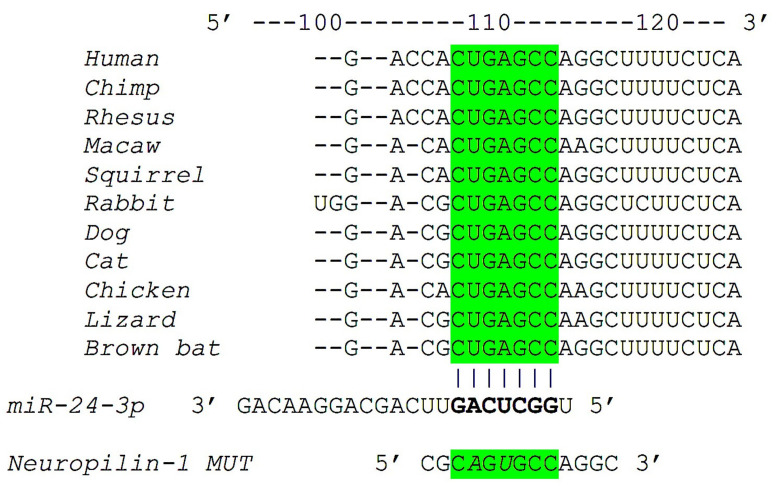
Identification of miR-24 as modulator of Neuropilin-1. Complementary nucleotides between the target region of Neuropilin-1 3′-UTR (in green) and hsa-miR-24-3p are highly conserved across different species.

To evaluate the effects of miR-24 on Neuropilin-1 gene transcription, we used a luciferase reporter containing the 3’-UTR of the predicted miRNA interaction site, both wild-type and mutated, in hBMECs cells. The mutant construct of Neuropilin-1 3′-UTR (Neuropilin-1 MUT, as shown in Figure 1), harboring a substitution of two nucleotides within the predicted miR-24 binding sites of Neuropilin-1 3′-UTR was obtained through means of the NEBaseChanger and Q5 site-directed mutagenesis kit (New England Biolabs, Ipswich, MA, USA) as we described [30,32].

We transfected hBMECs with the 3′-UTR reporter plasmid (0.05 μg) and miR-24 mirVana^TM^ mimics (ThermoFisher Scientific, Waltham MA, USA) or miR-24 miRIDIAN hairpin inhibitors (PerkinElmer, Waltham MA, USA), as well as a non-targeting negative control (scramble), all used at a final concentration of 50 nMol/L, using Lipofectamine RNAiMAX (ThermoFisher Scientific) [32]. Firefly and Renilla luciferase activities were measured 48 h after transfection, using a Luciferase Reporter Assay System (Promega, Madison, WI, USA), normalizing Firefly luciferase to Renilla luciferase activity [32]. 

Levels of miR-24 were measured via TaqMan miRNA assays (ThermoFisher Scientific), according to the manufacturer’s instructions, and normalized to the level of U6 as we previously described and validated [28,30]. Cellular expression of Neuropilin-1 was determined by RT-qPCR, as we described [28,30,33], normalizing to endogenous glyceraldehyde 3-phosphate dehydrogenase (GAPDH). Sequences of oligonucleotide primers (Merck KGaA, Darmstadt, Germany) are reported in Table 1.

### 2.4. Western Blot

Immunoblots were obtained as we previously described and validated [28,30,31] using the Odyssey system (LI-COR Biosciences, Lincoln, NE, USA); the intensity of bands was quantified using the FIJI software. 

The following antibodies were used: Neuropilin-1 (catalog number: #ST0530, Novus Biologicals, Bio-Techne, Minneapolis, MN, USA); Akt (catalog number: #sc-5298, Santa Cruz Biotechnology, Dallas, TX, USA); pAkt^Ser473^ (catalog number: #9271, Cell Signaling, Danvers, MA, USA); β Actin (catalog number: #ab8229, abcam, Cambridge, MA, USA).

### 2.5. Endothelial Permeability Assay

The in vitro permeability assay was performed as we previously described [34]. Briefly, hBMECs transfected with miR-24 mimic or miR scramble were grown on 0.4-mm fibronectin-coated (R&D Systems, Inc., Minneapolis, MN, USA) Transwell filters (Corning Inc., Corning, NY, USA). After 48 h, the medium in the upper well was replaced by FITC-dextran 70 kD (0.5 mg/mL in PB).

Cells were stimulated in the lower well with PBS alone or PBS containing 50 ng/mL VEGF-A_165_ (R&D Systems). The entity of endothelial permeabilization was determined measuring at 520 nm the fluorescence of Dextran that passed in the bottom chamber through the cell monolayer. 

### 2.6. Statistical Analysis

All data are expressed as means ± standard error of means (SEM). Statistical analyses were carried out using GraphPad 8 (Prism, San Diego, CA, USA). Statistical significance, set at *p* < 0.05, was tested using the two-way ANOVA followed by Tukey–Kramer multiple comparison test or the nonparametric Mann–Whitney U test, as appropriate. 

## 3. Results

### 3.1. Identification of miR-24 as a Specific Modulator of Neuropilin-1

A bioinformatic screening resulted in the identification of hsa-miR-24 as a highly conserved miRNA potentially capable of repressing Neuropilin-1 mRNA expression. The complementary nucleotides between the target region of Neuropilin-1 3’ untranslated region (3′-UTR) and miR-24 are evolutionarily highly conserved across different species, including humans, nonhuman primates, and rodents (Figure 1). 

### 3.2. Neuropilin-1 Is a Molecular Target of miR-24

**Figure 2:**
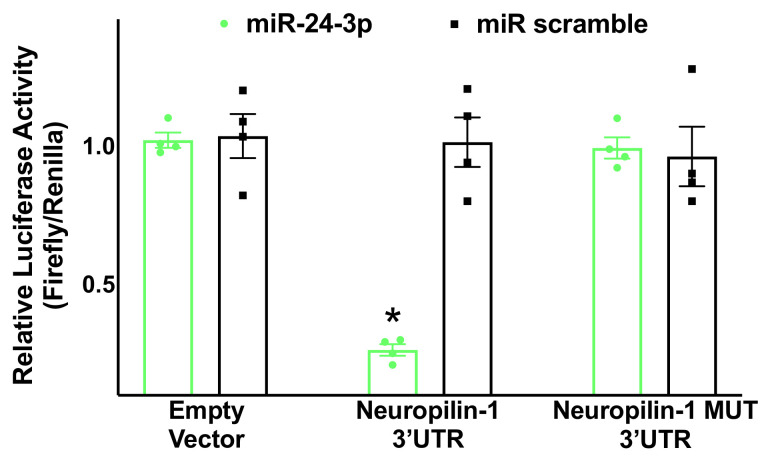
Validation of Neuropilin-1 targeting by miR-24. Luciferase activity was measured in hBMECs 48 h after transfection, using the vector without Neuropilin-1 3′-UTR (empty vector), the vector containing the wild-type Neuropilin-1 3′-UTR, and the vector containing a mutated Neuropilin-1 3′-UTR (Neuropilin-1 MUT); a non-targeting miRNA (miR scramble) has been employed as further control. Means ± S.E.M; * *p* < 0.05.

The proposed relationship was substantiated by an experimental validation of seed complementarity, confirming through a luciferase assay the interaction between miR-24 and the 3′-UTR of Neuropilin-1 in hBMECs (Figure 2).

### 3.3. miR-24 Regulates Neuropilin-1 Transcription Levels in Human Endothelial Cells

**Figure 3:**
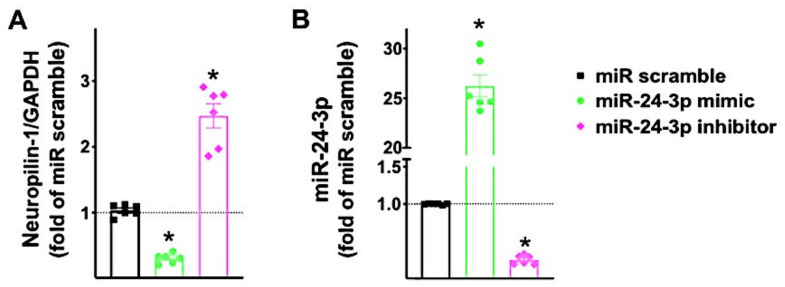
Neuropilin-1 expression in human brain endothelial cells is reduced by miR-24 and increased by miR-24 inhibitor. Neuropilin-1 mRNA levels (**A**) were measured in hBMECs transfected with miR-24 mimic, inhibitor, or scramble (negative control) for 48 h; miR-24 expression was assessed as well (**B**). Means ± S.E.M; * *p* < 0.05.

After having validated that miR-24 targets Neuropilin-1 3’UTR, we verified the effects of miR-24 mimic and miR-24 inhibitor on the transcription levels of Neuropilin-1 in hBMECs (Figure 3). These results were confirmed by immunoblot in terms of protein levels (Supplementary Figure S1).

### 3.4. miR-24 Regulates Neuropilin-1 Mediated Endothelial Permeability

Several investigators have demonstrated that Neuropilin-1 is involved in EC permeability [35,36,37]. To assess the functional role of miR-24 on Neuropilin-1 mediated endothelial permeability, we performed an in vitro permeability assay, following an experimental protocol that we have recently described [34]. 

**Figure 4:**
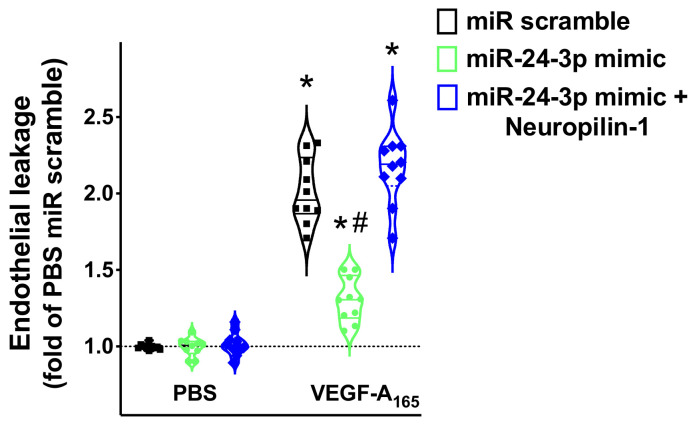
Targeting Neuropilin-1 by miR-24 significantly reduces endothelial permeability. Endothelial leakage triggered by VEGF-A_165_ (50 ng/mL) was measured in hBMECs transfected with miR-24-3p mimic, miR scramble, or combining miR-24-3p mimic and Neuropilin-1 overexpression; * *p* < 0.05 vs. PBS, # *p* < 0.05 vs. miR-scramble. PBS: Phosphate-buffered saline.

**Figure 5:**
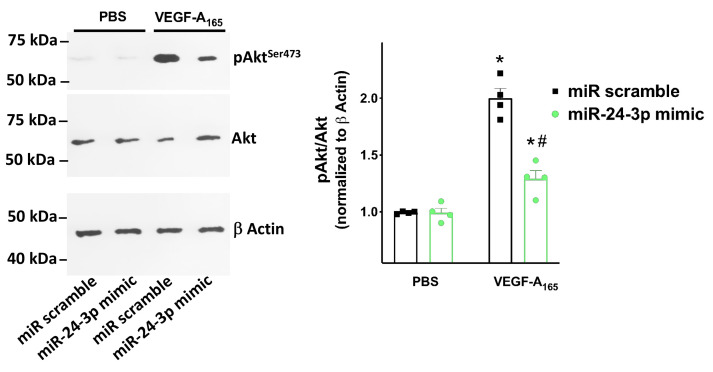
Targeting Neuropilin-1 by miR-24 significantly reduces Akt activation. Akt phosphorylation in Ser^473^ (representative immunoblots from 4 experimental assays) triggered by VEGF-A_165_ (50 ng/mL) was measured in hBMECs transfected with miR-24-3p mimic or miR scramble * *p* < 0.05 vs. PBS, # *p* < 0.05 vs. miR-scramble. PBS: Phosphate-buffered saline.

As shown in Figure 4, we found that miR-24 significantly reduced the permeability of hBMECs in response to VEGF_165_, an established agonist of Neuropilin-1 [4,38,39], and Neuropilin-1 overexpression rescued such an impaired response. Moreover, miR-24 mimic reduced VEGF_165_ induced Akt phosphorylation (Figure 5).

## 4. Discussion

In the present study, we have demonstrated for the first time that miR-24 directly targets the 3’UTR of Neuropilin-1. To the best of our knowledge, we also provide the first evidence of the actual expression of Neuropilin-1 in human brain endothelial cells. 

Our findings are consistent with previous research showing that Neuropilin-1 is expressed by pulmonary endothelial cells [40] and by tumor-associated vascular endothelial cells (TAVECs) [41]. Our data on miR-24 are in agreement with previous studies exploring the functional role of miR-24 in endothelial cells. Indeed, miR-24-3p has been shown to regulate angiogenesis in rodents, zebrafish embryos, and in diabetic patients by modulating endothelial function [42,43]. Additionally, miR-24 has been demonstrated to reduce endothelium-dependent inflammatory responses [44]. 

Neuropilin-1 is a transmembrane receptor that is abundant in the respiratory and olfactory epithelium and in olfactory-related regions such as the olfactory tubercles and para-olfactory gyri [45]. Two independent studies recently published in *Science* have demonstrated that Neuropilin-1 represents a crucial co-factor necessary for the entry of the severe acute respiratory syndrome coronavirus (SARS-CoV-2)—which causes the coronavirus disease 2019 (COVID-19)—in human cells [46,47]. The first one, led by Ludovico Cantuti-Castelvetri [46] has shown that Neuropilin-1 significantly potentiates SARS-CoV-2 infectivity; the second one, led by James L. Daly, has proven via biochemical approaches and x-ray crystallography that the cleaved Spike protein of SARS-CoV-2 directly binds Neuropilin-1 [47]. 

Mounting evidence has shown that SARS-CoV-2 can directly target endothelial cells [48,49,50,51,52,53,54,55,56,57], an aspect initially reported by our group in March 2020 by simply observing the systemic manifestations in COVID-19 patients [48,49,58], and later corroborated by autoptic findings [59,60,61,62,63] and by the analysis of amputation specimens [64]. Intriguingly, brain endothelial cells show a distinct pro-inflammatory response when exposed to SARS-CoV-2 spike protein subunits [65] and infected vascular endothelial cells have been shown to spread SARS-CoV-2 to glial cells in the central nervous system [66]. Furthermore, COVID-19 has been associated with a wide spectrum of neurological symptoms and Neuropilin-1 has been proposed as a key factor in the neurological manifestation of COVID-19 by enhancing the entry of SARS-CoV-2 into the brain [66,67,68,69,70,71].

The most studied ligands of Neuropilin-1 are vascular endothelial growth factor (VEGF), semaphorins, complement split products, and furin-cleaved substrates [1,2,3,4,72]. Interestingly, augmented VEGF levels have been reported in bronchial alveolar lavage fluid from COVID-19 patients [73] and asymptomatic COVID-19 have lower serum VEGF levels compared to symptomatic patients [74]. In line with these observations, the interaction between Neuropilin-1 and VEGF has been recently shown to be implied in nociception [75].

Neuropilin-1 could also be involved in the relationship between COVID-19 and diabetes mellitus. Various studies have demonstrated that severe COVID-19 disproportionately affects patients with diabetes [76,77]. Of note, among the proposed SARS-CoV-2 cell-entry and amplification factors assessed in a cryopreserved human diabetic kidney single-nucleus RNA sequencing dataset [78], only Neuropilin-1 was found to be significantly upregulated [46]. In agreement with these reports, hyperglycemia has been shown to downregulate miR-24 expression in plasma and tissue and knocking miR-24 down in mice leads to increased expression and secretion of von Willebrand factor in endothelial cells, accompanied by a significantly enhanced platelet tethering [79], thereby suggesting a pathophysiologic role for this miRNA in the thromboembolic complications described in COVID-19 [49,80,81,82]. 

In addition to endothelial cells, Neuropilin-1 is expressed in immune cells, including T cells, B cells, macrophages, dendritic cells, and mast cells, where it regulates development, migration, recruitment, and communication between different immune cells [83]. Despite emerging evidence for the immune regulatory functions of Neuropilin-1, its exact molecular pathways remain not fully understood. Nevertheless, it is likely that Neuropilin-1 could be also involved in the cytokine storm and the subsequent hyper-inflammatory state observed in COVID-19 patients [84,85,86], although further dedicated studies in this sense are necessary.

Our study should be interpreted in light of some limitations. For instance, we only performed in vitro experiments testing the association between miR-24 and Neuropilin-1 mRNA, and we did not verify the actual effects of miR-24 on SARS-CoV-2 infection. Since most of the findings were obtained using exogenously expressed miRNAs, further studies are required to evaluate the translational potential of our results. Nonetheless, our findings are consistent with the observation of miR-24 expression in endothelial cells [42,43,44,87] and its roles as a regulator of various cerebrovascular phenomena, including angiogenesis in gliomas [88,89] and vasospasm following subarachnoid hemorrhage [90]. The study also has some strengths, including the fact that the 3′-UTR of Neuropilin-1 that is targeted by miR-24 is highly conserved among species, from primates to rodents. 

In conclusion, our data show for the first time that Neuropilin-1 is a direct target of miR-24 in human brain endothelial cells. 

## Figures and Tables

**Table 1:** Sequences of oligonucleotide primers and product sizes.

*Gene*	Primer	Sequence (5′–3′)	Amplicon (bp)
*Neuropilin-1*	Forward	CCA CAG TGG AAC AGG TGA TG	114
	Reverse	ACA CAC ACA GGC GTT AGC TG	
*GAPDH*	Forward	GGC TCC CTT GGG TAT ATG GT	94
	Reverse	TTG ATT TTG GAG GGA TCT CG	

GAPDH: glyceraldehyde 3-phosphate dehydrogenase.

## Data Availability

The data herein presented are available in this article and Supplementary Material.

## Supplementary Materials

The following are available online at https://www.mdpi.com/2311-553X/7/1/9/s1, Figure S1: Neuropilin-1 protein expression is regulated by miR-24-3p.
