# Supplementary material for: miR-24 Targets the Transmembrane Glycoprotein Neuropilin-1 in Human Brain Microvascular Endothelial Cells
Source: Noncoding RNA. 2021 Feb 2;7(1):9. doi: 10.3390/ncrna7010009 (PMC7931075; doi:10.3390/ncrna7010009)
Supplement: Supplementary file 1 [file ncrna-07-00009-s001.pdf]

Supplementary Material

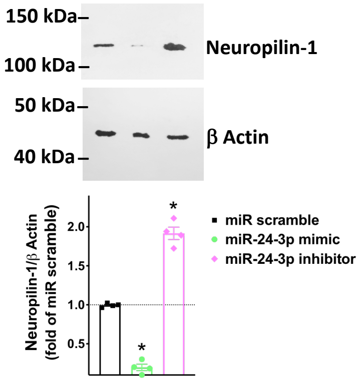

Supplementary Figure S1. Neuropilin-1 protein expression is regulated by miR-24-3p. Neuropilin-1 protein levels were assessed by immunoblot in human brain microvascular endothelial cells (hBMECs) 48 hours after transfection with miR-24 mimic, miR-24 inhibitor, and miR-scramble (negative control). Representative immunoblots from four independent experiments are shown. Data are means ± S.E.M. *:$p<0.05$.
